# 10 Years of Preparedness by the Radiation Injury Treatment Network

**DOI:** 10.1007/s11899-017-0360-7

**Published:** 2017-01-31

**Authors:** Cullen Case

**Affiliations:** Radiation Injury Treatment Network, National Marrow Donor Program, 500 N. 5th Street, Minneapolis, MN 55401 USA

**Keywords:** Radiological, Emergency, Disaster, Marrow toxic injuries, Preparedness, Transplant

## Abstract

The Radiation Injury Treatment Network (RITN) began in 2006 with the ambitious vision to provide a resource to help with the surge of casualties following a mass casualty incident with marrow toxic injuries. Through the efforts of the National Marrow Donor Program and American Society for Blood and Marrow Transplantation with the support of the Office of Naval Research, the initial 13 hospitals and cancer centers have grown to 76, training over 13,500 hospital staff and conducted, funded, and supported 580 disaster exercises testing preparedness. After a decade, there is more to do, but much laudatory work has been accomplished.

“the specter of nuclear terrorism still threatens us all.”

-President Barack Obama

June 2, 2016 address to the US Air Force Academy [1]

The Radiation Injury Treatment Network (RITN) began with the ambitious vision to provide a resource to help with the surge of casualties following a mass casualty incident with marrow toxic injuries. This continues to be a lofty goal that the RITN strives for, but the gap between where we were following the tragedy of 9/11 and today is significantly smaller. The National Marrow Donor Program (NMDP) has had the great fortune to have a grant from the Office of Naval Research to conduct research to improve outcomes from hematopoietic transplantation. As part of this grant, there has been a sub-line item to conduct contingency planning in preparedness for a national disaster. This effort was half-heartedly fulfilled for a few years then the efforts were greatly accelerated following 9/11. The NMDP began by holding focus sessions with pre-imminent Hematology and Oncology physicians that were part of the NMDP’s network of transplant centers. These meetings led to the close partnership with the American Society for Blood and Marrow Transplantation (ASBMT) which broadened the audience and helped to continue to engage those who would be involved in a response. These focus groups, surveys, and meetings culminated in the formation of the Radiation Injury Treatment Network in 2006 with the commitment of 13 blood and bone marrow transplant units at NMDP affiliated hospitals/cancer centers.

Hematology and oncology teams have extensive expertise in caring for patients that have been purposefully exposed to high levels of ionizing radiation in preparation for hematopoietic stem cell transplantation. The resulting complications can be directly correlated to the care needed for a radiation casualty suffering from Acute Radiation Syndrome resulting from exposure to an ionizing radiation. A radiological disaster could be as catastrophic as the detonation of an improvised nuclear device (terrorist nuclear bomb) or a less devastating incident such as a radiological exposure device (a radiological source placed in a public space), a radiological dispersal device (a.k.a. a dirty bomb where a conventional explosive spreads radiological material), a nuclear power plant accident, or a mustard agent incident. Regardless of the incident, there will be casualties that require specialized supportive care to salvage their damaged hematopoietic systems.

From these small beginnings, the group would debate the focus, vision, and even the name of what would become the RITN, culminating in 2006 with the signing of the first 13 contracts. This was no easy task as initially the language stated the hospitals would accept patients from a radiological disaster, which the hospital administration and legal staff expressed concern over. After going a few rounds of review, with each hospital, language that was strong enough to show commitment yet flexible enough to appease the lawyers solidified. The sales pitch to new institutions was a repeat of selling the pros of participation; imagine asking a senior administrator at a hospital if they would like to join a group of hospitals preparing to receive casualties from the detonation of a terrorist’s nuclear device. Needless to say there was some pushback. However, fortunately for RITN the physicians we worked with were passionate and persuasive. Since those humble beginnings RITN has grown into a conglomeration of 76 cancer treatment centers, hospitals, blood donor centers, and cord blood banks as of late 2016.

The hospitals, cancer centers and the physicians that led the charge at the initial cohort of cancer centers that joined RITN should be recognized for their efforts:

1. Barnes-Jewish Hospital at Washington University School of Medicine (John DiPersio, M.D.)
2. Cincinnati Children’s Hospital Medical Center (Stella Davies, M.D.)
3. City of Hope National Medical Center (Auayporn Nadamanee, M.D.)
4. Dana-Farber/Partners Cancer Care (Joseph Antin, M.D.)
5. Duke University Medical Center (Joanne Kurtzberg, M.D.)
6. M.D. Anderson Cancer Center (Marcos deLima, M.D.)
7. Memorial Sloan-Kettering Cancer Center (Nancy Kernan, M.D.)
8. Presbyterian/St. Luke’s Medical Center (Mark Brunvand, M.D.)
9. Seattle Cancer Care Alliance (Ann Woolfrey, M.D.)
10. Stanford Hospital and Clinics (Keith Stockerl-Goldstein, M.D.)
11. Texas Children’s Hospital (Robert Krance, M.D.)
12. University of Minnesota BMT Program (Margaret MacMillan, M.D.)
13. University of Pennsylvania Medical Center (David Porter, M.D.)

**a complete list of current RITN hospitals by location can be found at www.RITN.net/About

As stated, these 13 have grown to 76 which can be seen in Fig. 1: location of RITN centers.

**Fig. 1:**
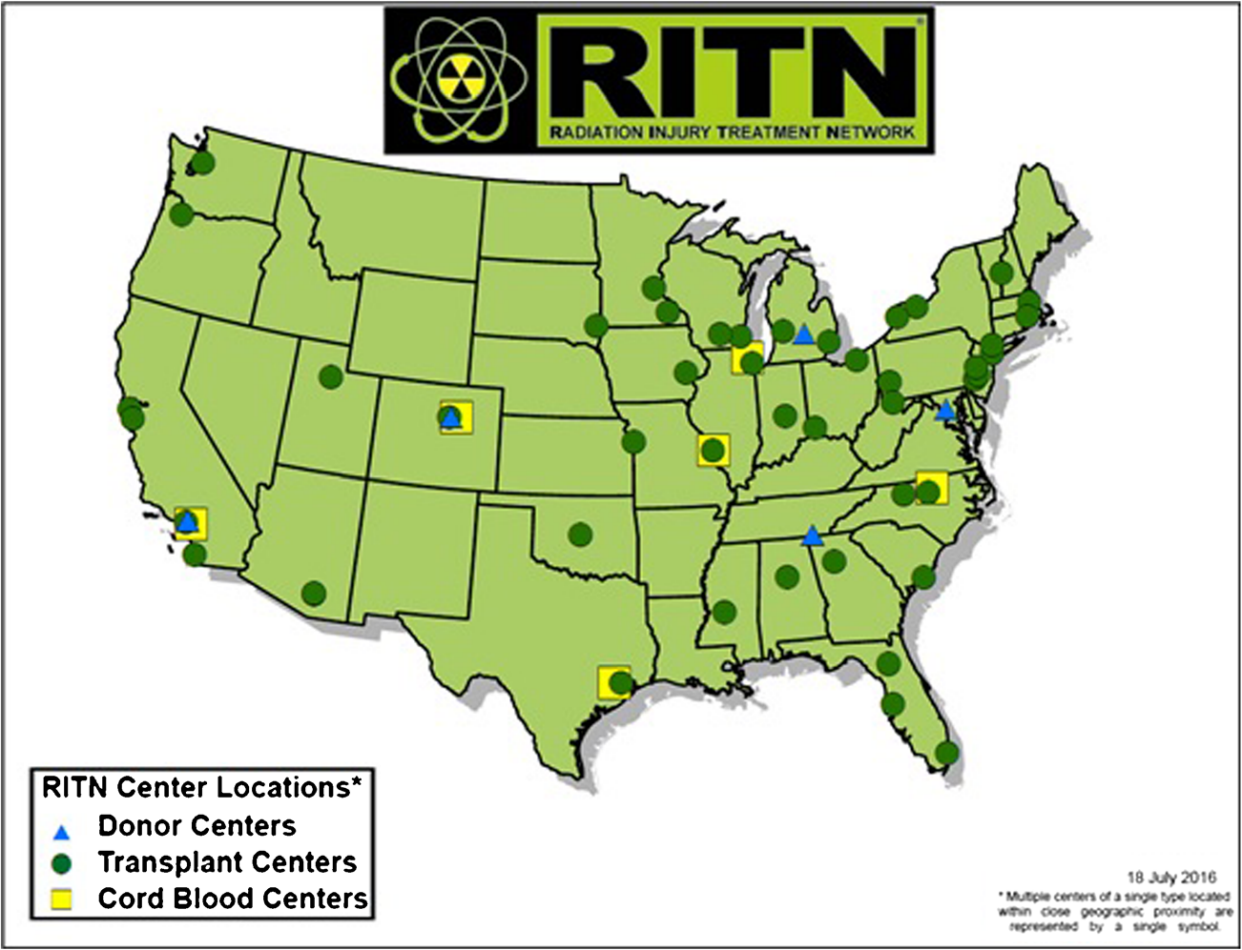
Location of RITN centers

To encourage participation, we appealed to each hospital’s sense of greater good as well as offered a token stipend to offset some of the costs, this grant did not cover the cost of staff time fully but at least eased the sting. To help with this effort, the funds were provided through a fee for service agreement to reduce the restrictions on how they could be used. However, to ensure that we were “getting our monies worth,” we began assigning annual tasks. These included the development of a standard operating procedure, incorporation of the RITN acute radiation syndrome treatment guidelines, training, conducting an exercise (emergency drill), and testing the satellite telephone and emergency calling card (Government Emergency Telecommunications Service) that were issued to each center. To ease this burden, we developed the training and exercise so that each center could readily apply them with minimal effort. We still ask each center to accomplish similar tasks each year; however, we have added a dozen training options so that it does not get stale and broadens the perspective on impact on a local center in the event of a nuclear disaster. This initiative grew to include training, emergency preparedness exercises, and regular workshops bringing world-renowned experts together to plan and prepare for a catastrophe, that everyone hopes will never happen.

RITN has developed and made available to the public many of the resources developed over the past 10 years to improve preparedness including training materials, disaster exercises/drills materials, treatment guidelines, medical order sets for adult and pediatric patients, and referral guidelines (Fig. 2). All of these are available on the RITN website for open use by the public; providing recognition is given to RITN as the source. Since 2006, the RITN has assisted in the training of over 13,500 medical and hospital support staff on radiological-based training. All of these materials are available for use and review on our public website [2] including links to our free web-based training. MS PowerPoint slides are available to conduct training as needed to extend the effort.

**Fig. 2:**
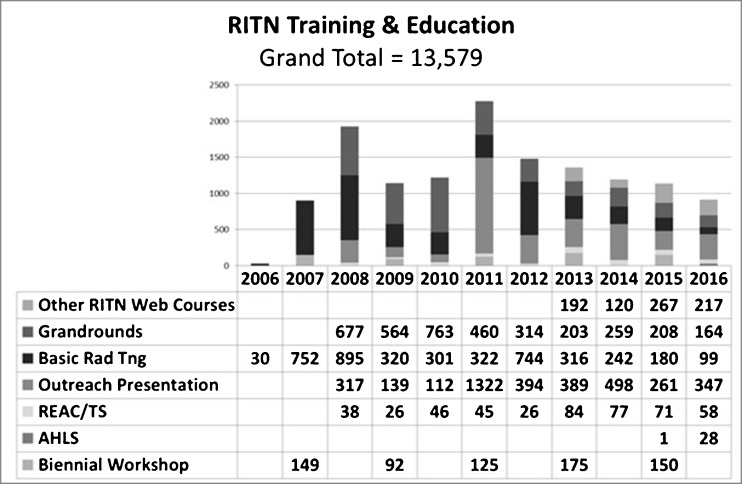
RITN training and education

Since the inception of RITN, exercises have been a part of the annual requirements for each hospital. Each year, RITN would develop a tabletop exercise, where a disaster scenario would be reviewed by appropriate staff at the hospital and, when appropriate, with the emergency preparedness teams of the community. They would then provide responses to questions on what they would have done in said situation. These efforts grew to include functional exercises, where portions of the response plans are acted out, to full-scale exercises where mock casualties with moulage wound makeup, ambulances, and aircraft are mobilized for a close to realistic situation as possible. All told, over 580 exercises have been coordinated or funded by RITN from 2006 to 2016 (Fig. 3). RITN does not want those who are planning an exercise to duplicate previous efforts, so in addition to the after action reports from exercises, explaining the lessons learned and gaps identified, the full exercise planning and execution documents are posted for use on our website for general review [3].

**Fig. 3:**
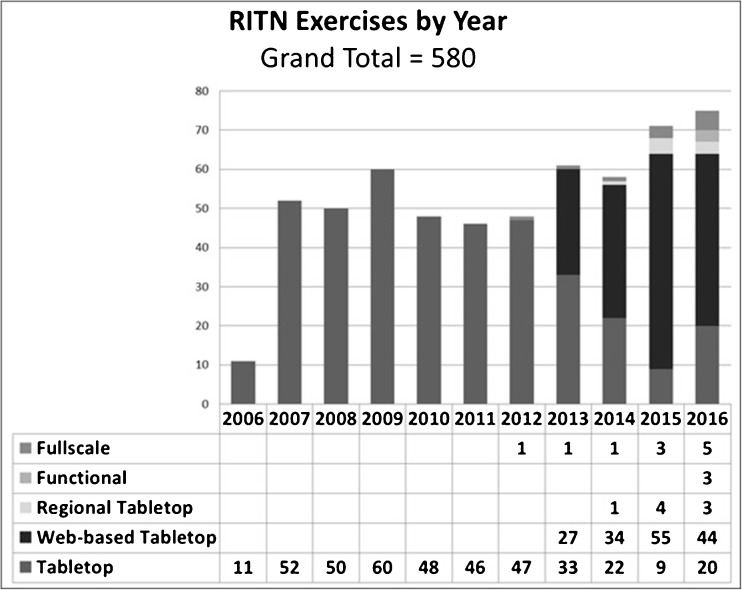
RITN exercises by year

Hospitals preparing for a mass casualty incident require more than training and exercises. Just-in-time tools are often requested, and we have tried to meet these needs through the development of many resources. These include the RITN ARS Treatment Guidelines, RITN Hospital Referral Guidelines, RITN Concept of Operations, the Adult and Pediatric Medical Ordersets (which were developed in coordination with the Radiation Emergency Medical Management (REMM) website) for implementation of a standard approach to patient care for those individuals who might experience an acute radiation illness.

RITN has come a long way; we still see many challenges ahead; need for continued growth of the network, how to engage distant unused medical capacity through telemedicine, and increasing awareness within the medical and public health community, as well as many other local issues that are identified during the exercises we support. However, based on the great success we have seen to date, we are confident that the Radiation Injury Treatment Network will continue to step up to the challenge presented.

RITN’s mission and goals are as follows[4]:

The Radiation Injury Treatment Network® (RITN) provides comprehensive evaluation and treatment for victims of radiation exposure or other marrow toxic injuries.

Many of the casualties with radiation injury will be salvageable but require outpatient and/or inpatient care. Recognizing this, the US National Marrow Donor Program (NMDP), US Navy and American Society for Blood and Marrow Transplantation (ASBMT) collaboratively developed RITN, which comprises of medical centers with expertise in the management of bone marrow failure, stem cell donor centers and umbilical cord blood banks across the US.

The goals of RITN are as follows:

1. *to develop treatment guidelines for managing hematologic toxicity among victims of radiation exposure*,
2. *to educate health care professionals about pertinent aspects of radiation exposure management*,
3. *to help coordinate the medical response to radiation events, and*
4. *to provide comprehensive evaluation and treatment for victims at participating centers*.

Since RITNs inception, it has been fortunate to have many partners including the support of the Office of Naval Research, Department of Health and Human Services Health Resources Services Administration, Department of Health and Human Services Assistant Secretary for Preparedness and Response, and the Radiation Emergency Medical Management website operated by the National Library of Medicine as well as many other prominent supporters including the National Association of County and City Health Officials (NACCHO), Association of State and Territorial Health Officials (ASTHO) National Alliance for Radiation Readiness (NARR), European Society for Blood and Marrow Transplantation (EBMT), World Health Organization-Radiation Emergency Medical Preparedness and Assistance Network, Radiation Emergency Assistance Center and Training Site (REAC/TS), and many others.
